# Physiological Root Resorption of Deciduous Teeth: An ATR-FTIR Approach

**DOI:** 10.3390/jcm14010048

**Published:** 2024-12-26

**Authors:** Giulia Orilisi, Alessia Cosi, Flavia Vitiello, Chiara Santoni, Valentina Notarstefano, Elisabetta Giorgini, Giovanna Orsini, Domenico Tripodi

**Affiliations:** 1 Department of Clinical Sciences and Stomatology (DISCO), Università Politecnica delle Marche, 60126 Ancona, Italy; g.orilisi@pm.univpm.it (G.O.); f.vitiello@pm.univpm.it (F.V.); 2 Department of Medical, Oral and Biotechnological Sciences, University “G. D’Annunzio” of Chieti-Pescara, Via dei Vestini 31, 66100 Chieti, Italy; alessiacosiac@gmail.com (A.C.); tripodi@unich.it (D.T.); 3 Department of Life and Environmental Sciences, Università Politecnica delle Marche, Via Brecce Bianche 12, 60131 Ancona, Italy; c.santoni@pm.univpm.it; 4 Department of Bioscience and Technology for Agriculture, Food and Environment, University of Teramo, Campus Aurelio Saliceti, Via Balzarini 1, 64100 Teramo, Italy; v.notarstefano@univpm.it

**Keywords:** root resorption, deciduous teeth, ATR-FTIR spectroscopy, dental pulp biological processes

## Abstract

**Background**: The study exploited, for the first time, Attenuated Total Reflectance-Fourier Transform-InfraRed (ATR-FTIR) spectroscopy on human dental pulps at different timings of root resorption (RR) to deepen the biological mechanisms occurring in deciduous teeth (De) during their replacement with permanent ones. **Methods**: N:36 dental pulps from sound De were divided into the following: G0 (no RR); G1 (RR less than 1/3 of root length); G2 (RR not exceeding 2/3 of root length); and G3 (RR more than 2/3 of root length). Samples were analyzed by ATR-FTIR, and the spectral data were submitted to univariate (One-way ANOVA and Tukey’s multiple comparison tests; statistical significance set at *p* < 0.05) and multivariate (Principal Component Analysis, PCA) analyses. **Results**: PCA displayed good discrimination among groups, ascribable to: (i) the intensity of the peaks of nucleic acids (~1715 cm^−1^, ~1237 cm^−1^, ~964 cm^−1^, and ~815 cm^−1^) and carbohydrates (~1159 cm^−1^) which increased from G0 to G3 (*p* < 0.05); (ii) the relative amount of lipids which decreased from G0 to G3 (*p* < 0.05); and (iii) the intensity of the peaks at ~1014 cm^−1^, and ~875 cm^−1^ (phosphates and carbonates in hydroxyapatite), which decreased from G0 to G3 (*p* < 0.05). **Conclusions**: This study confirmed ATR-FTIR as a reliable and quick technique for the characterization of the dental pulp and highlighted a correlation between specific molecular changes in the dental pulp of deciduous teeth and different RR stages, shedding new light on this process and paving the way for future research, which could improve the clinical management of the primary dentition.

## 1. Introduction

The root resorption (RR) and exfoliation of deciduous teeth (De) represent a complex physiological process that occurs in a specific period of childhood to provide the space for the eruption of the permanent successors [1]. This process, named rizalysis, includes not only the resorption of the hard tissues of De, such as dentin and cementum, but also the elimination of soft tissues, including the pulp and the periodontal ligament [1].

Understanding in-depth the physiological root resorption of De is essential for clinicians to choose the more predictable and appropriate treatment. Unfortunately, to date, the biochemical mechanisms involved are not yet clear, even if researchers have predicted and summarized the molecular and histological events occurring during this physiological process [2,3]

Despite some conflicting results reported in the literature [4,5], a prominent role seems to be played by dental pulp cells [6]. In fact, one of the earliest changes observed by histological analysis was the activation of odontoclasts, which are responsible for the degradation of cementum and dentin [7]. Odontoclast recruitment and activity are also modulated by inflammatory cells, which represents another fundamental component during RR [8]. Indeed, the physiological RR of De is regulated by multiple inflammatory cytokines and key transcription factors through different signaling pathways, mediating monocyte-macrophage lineage to form osteo/odontoclast [6]. The inflammatory microenvironment leads to morphological changes in the dental pulp, and odontoblasts begin the degradation [2]. Concomitant with inflammatory infiltration, vascular changes occur, and the increased blood supply supports the metabolic demands of the resorptive process and facilitates the recruitment of immune and reparative cells [4,9]. Despite these insights, questions remain about both the precise mechanisms underlying these histological modifications and the relation between the degree of RR and macromolecular changes.

Among the non-conventional analytical techniques used in the biomedical field, an interesting role is played by Fourier Transform InfraRed spectroscopy (FTIR). This is a non-destructive, highly sensitive, and label-free technique valuable for characterizing the chemical composition of biological samples, including tissues, cells, and fluids [10]. The principle FTIR spectroscopy relies on is that the chemical bonds within a material/compound can absorb the electromagnetic radiation in the infrared region, undergoing a vibrational transition. Each transition can be observed and expressed in terms of wavenumbers (cm^−1^) on the *x*-axis of a bidimensional graph, while on the *y*-axis, the intensity of the absorption is shown. This is an absorbance IR spectrum, in which each maximum corresponds to a vibration or a group of vibrations of a chemical bond. Interestingly, the frequencies absorbed by each bond depend on the strength of the bond and the atoms involved. Hence, each chemical bond displays only specific absorptions/peaks that can make it distinguishable. Since IR peaks can sometimes overlap, the absorbance IR spectrum can be transformed into the Second Derivative mode; this procedure allows the detection of all the underlying peaks, which are shown as minima. Hence, FTIR analysis of biological samples provides not only the macromolecular fingerprint of the analyzed sample, including the composition of lipids and proteins and the characterization of the carbohydrates and phosphates in glycosylated compounds and nucleic acids, but also highlights changes due to specific biological processes or treatments [10,11].

Within FTIR, Attenuated Total Reflectance (ATR) is a very interesting sampling methodology suitable for the analysis of homogeneous solid and liquid samples, such as biological fluids and cells, without further preparation. ATR-FTIR spectra are independent of the depth of penetration of the IR energy into the sample [12]; in fact, it exploits the total internal reflection to produce an evanescent wave that penetrates the sample for only a few microns, providing valuable molecular information. In this light, ATR-FTIR is considered a powerful and fast analytical tool able to highlight macromolecular alterations in cellular samples due to specific biological processes and drug treatments [13,14,15].

Based on this evidence and due to the lack of information regarding the chemical modifications in De dental pulp during the RR process, in the present study ATR-FTIR spectroscopy was exploited, for the first time, to detect possible differences in terms of the composition and relative amount of proteins, lipids, carbohydrates, and nucleic acids in relation to different timings of RR. The null hypothesis of this study was that the degree of RR does not influence the macromolecular composition of the dental pulp in deciduous teeth.

## 2. Materials and Methods

### 2.1. Samples Collection and Classification

N. 36 deciduous teeth were obtained from healthy children, aged between 5 and 10 years, who needed dental extraction for therapeutic or clinical purposes, both at the Section of Pediatric Dentistry of the University “G. D’Annunzio” Chieti-Pescara and at the Department of Clinical Sciences and Stomatology of the Università Politecnica delle Marche (Ancona, Italy). The patient’s parents signed an informed consent, according to the Local Ethical Committee guidelines and the WMA–Declaration of Helsinki (2018) [16], fully aware that their hard dental tissues, as a discard of the surgical procedures, would be used for research purposes. Teeth diagnosed with any pathology other than attrition and superficial enamel caries were excluded from the study. Immediately after the extraction, samples were immersed in an ultrasonic bath with distilled water for 3 min in order to remove blood and biological remains. Then, an occlusal cavity was performed on each tooth using a conic diamond burr, and the pulp tissue was gently retrieved by a sterile endodontic file (K-File #8, Dentsply Sirona, Charlotte, NC, USA). Extra caution was taken to avoid the contamination of the files. Dental pulp tissues were fixed with 4% paraformaldehyde phosphate buffer solution (PFA; Wako Pure Chemical Industries Ltd., Osaka, Japan) for 20 min and then were kept at −20 °C in Eppendorf tubes with deionized water until the analyses were performed.

The degree of root resorption for each extracted tooth was established following a well-known protocol reported in the literature [4,17]. More in detail, the vertical dimension, defined as the distance between the enamel-cement junction, and the point of RR at the deepest level [2] was measured by using an electronic millimeter caliper (Digimatic Calliper, Mitutuyo, Ltd., Hampshire, UK). The percentage of the total root length that had undergone the RR was calculated according to Kramer and Ireland’s published norms [5].

This analysis was used to divide all the samples into the following groups according to the degree of RR (see Table 1): G0, N. 7 intact De teeth, no RR occurred; G1, N. 9 De with RR less than 1/3 of the root length; G2, N. 10 De with RR has not exceeded 2/3 of the root length; and G3, N. 10 De with RR was more than 2/3 of the root length).

### 2.2. ATR-FTIR Measurements and Data Analysis

ATR-FTIR measurements were performed by using a Bruker Invenio-R interferometer equipped with a Platinum ATR accessory mounting a diamond crystal and a Deuterated TriGlycine Sulfate detector (Bruker Optics, Ettlingen, Germany).

The following procedure was followed. Samples were deposited onto the diamond crystal and gently pressed to obtain good adhesion to the surface of the crystal. For each sample, three ATR-FTIR spectra were collected at room temperature in the 4000–600 cm^−1^ range (128 scans, 4 cm^−1^ spectral resolution). Before each sample acquisition, the spectrum of the background was collected on the clean diamond crystal under the same conditions. Raw spectra were corrected for atmospheric carbon dioxide and water vapor (Atmospheric Compensation routine), vector normalized in the whole spectral range (Vector Normalization routine), interpolated in the 3050–750 cm^−1^ range (Cut routine), and then two-point baseline linear fitted (Baseline Correction routine) (OPUS 7.5 software, Bruker Optics, Ettlingen, Germany). For each sample, the average spectrum was then calculated.

Pre-processed IR spectra were interpolated in the 1800–900 cm^−1^ spectral range and analyzed by Principal Component Analysis (PCA), both in Absorbance and Second Derivative modes (Origin PRO 2018 software).

The same pre-processed spectra were also submitted to the Curve Fitting procedure under the 3050–2800 cm^−1^ and 1800–750 cm^−1^ ranges. This procedure lets us detect the exact position and integrated area of all the underlying peaks under a specific spectral interval. The number and position (expressed as wavenumbers) of the underlying peaks were identified by second derivative minima analysis and fixed during the fitting procedure with Gaussian functions (GRAMS/AI 9.1, Galactic Industries, Inc., Salem, NH, USA). The areas obtained were used to calculate specific spectral parameters, described in the Results section.

### 2.3. Statistical Analysis

IR data were presented as mean ± S.D. and statistically analyzed by One-way ANOVA and Tukey’s multiple comparison test by using the GraphPad Prism software version 8.00 (GraphPad Software, San Diego, CA, USA). Statistical significance was set at *p* < 0.05.

The sample size calculation was performed to have a minimal difference between the intensity of the analyzed peaks between groups. The α value was determined as 0.05, while the power of the test was 0.95. For the statistical calculation, G-power sample size 3.1 software calculation was used.

## 3. Results

**Figure 1:**
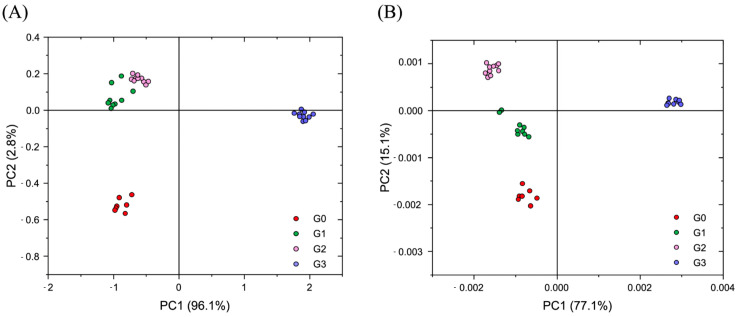
Principal Component Analysis was performed on G0 (red circles), G1 (green circles), G2 (pink circles), and G3 (violet circles) spectral populations. PCA score plots are displayed both on (**A**) absorbance (Abs) and (**B**) second derivative (DII) spectra. (**A**) As regards spectra in Absorbance mode, a complete separation is observed between G0 and G3 samples (PC1 axis, explained variance 96.1%), while G1 and G2 are almost overlapping but segregated with respect both to G0 and G3 (PC2 axis, explained variance 2.8%). (**B**) As regards spectra in the Second Derivative mode, a complete separation is displayed among all groups; G0, G1, and G2 are segregated with respect to G3 (PC1 axis, explained variance 77.1%), while G0 and G1 are separated with respect to G2 and G3 (PC2 axis, explained variance 15.1%).

Principal Component Analysis (PCA) was first employed as an unsupervised statistical approach for the observation of possibly correlated changes among the dental pulps collected from De at different degrees of RR. The analysis was performed on both Absorbance (Abs) and Second Derivative (DII) spectra to better highlight differences in the spectral profiles of the different spectral populations. More in detail, as regards Abs spectra (Figure 1A), complete segregation was observed between G0 and G3 (PC1 axis, explained variance 96.1%), while G1 and G2 were almost overlapping but segregated with respect both to G0 and G3 (PC2 axis, explained variance 2.8%). The PCA scores plot of DII spectral populations (Figure 1B) showed better segregation among all groups; in fact, the PC1 axis separated G0, G1, and G2 spectra from G3 ones (explained variance 77.1%), while PC2 discriminated between G0 and G1 spectra and G2 and G3 ones (explained variance 15.1%).

**Figure 2:**
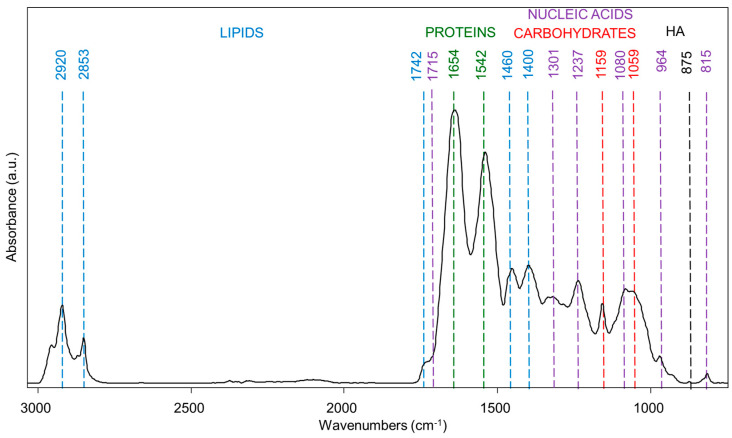
The IR spectrum of a representative dental pulp collected from a sound deciduous tooth. The spectrum is shown in absorbance mode in the 3050–750 cm^−1^ spectral interval. The position of the main absorption bands is reported, together with the corresponding organic and inorganic components (light blue, LIPIDS; green, PROTEINS; violet, NUCLEIC ACIDS; red, CARBOHYDRATES, and black, hydroxyapatite HA).

In Figure 2, the spectral profile of a representative sample of dental pulp collected from a sound De is shown. The IR spectrum is reported in absorbance mode in the 3050–750 cm^−1^ interval, and the most relevant peaks, attributable to lipids, carbohydrates, proteins, and nucleic acids, which are the most common components of biological samples, are evidenced [15,18]. More in detail, the bands at ~2920 cm^−1^ and ~2853 cm^−1^ are diagnostic for lipids, as they are associated with the asymmetric and symmetric stretching vibrations of CH_2_ moieties in lipid alkyl chains; some information about lipids can also derive from the bands at ~1460 cm^−1^ and ~1400 cm^−1^, which are likely due to bending vibrations of the same alkyl chains [19]; finally, the band at ~1742 cm^−1^ is typical of the carbonyl bond in triglycerides and fatty acids [20]. Proteins are characterized by two intense bands, named Amide I and II, centered at ~1654 cm^−1^ and ~1542 cm^−1^. These are convoluted bands whose analysis provides relevant information on proteins’ secondary structure, including the presence and relative amount of a and triple helices, b sheets, as well as random coil components [21]. Carbohydrates show two bands at ~1157 cm^−1^ and ~1059 cm^−1^, attributable to the stretching vibrations of the C-O bond and CH_2_OH moiety [15]. Several bands diagnostic for nucleic acids are also evident, such as the band at ~1301 cm^−1^ related to the bending of the N-H bond, the bands at ~1237 cm^−1^ and ~1080 cm^−1^ assigned to the asymmetric and symmetric stretching vibrations of phosphate groups both in RNA and DNA [22], and the bands at ~964 cm^−1^ and ~815 cm^−1^ due to vibrations of the DNA backbone [23]. Finally, the band at ~875 cm^−1^ is related to inorganic phosphate groups in the hydroxyapatite [24].

**Figure 3:**
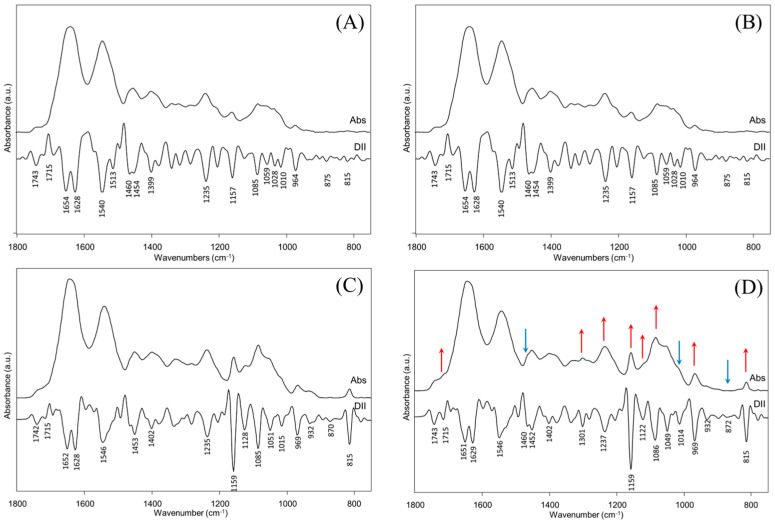
Average IR spectra of dental pulps collected from deciduous teeth at different timing of root resorption: (**A**) G0; (**B**) G1; (**C**) G2, and (**D**) G3 experimental groups (red and blue arrows indicate the bands that arise or decrease in G3 respect to G0). IR spectra are shown in absorbance (Abs) and second derivative (DII) modes in the 1800–750 cm^−1^ spectral interval. The position of the main absorption bands is reported below DII spectra. Red and blue arrows over peaks indicate the bands which change RR: red ones display an increase in absorbance, while blue ones a decrease.

Due to the presence of convoluted bands, the profile of which derives from the overlap of multiple vibrational transitions, the region going from 1800 cm^−1^ to 750 cm^−1^, which includes the main absorptions of both the organic and inorganic components, was separately analyzed in Absorbance and Second Derivative modes (Figure 3). The position of the main peaks identified also by the Curve Fitting procedure, together with the corresponding vibrational mode and the biological meaning, are reported in Table 2.

The analysis of the spectra in Figure 3 let evidence some differences among groups (highlighted by blue and red arrows): (i) as regards nucleic acids, the peaks at ~1715 cm^−1^, ~1301 cm^−1^, ~1237 cm^−1^, ~1122 cm^−1^, ~1086 cm^−1^, ~964 cm^−1^, and ~815 cm^−1^, attributable to DNA and RNA, are more intense in G2 and G3 with respect to G0 and G1; (ii) a similar behavior was displayed by the band at ~1159 cm^−1^, assigned to carbohydrates; (iii) conversely, the bands at ~1014 cm^−1^, and ~1460 cm^−1^, ~875 cm^−1^, assigned respectively to inorganic phosphates and carbonates in hydroxyapatite, decrease in intensity going from G0 to G3.

**Figure 4:**
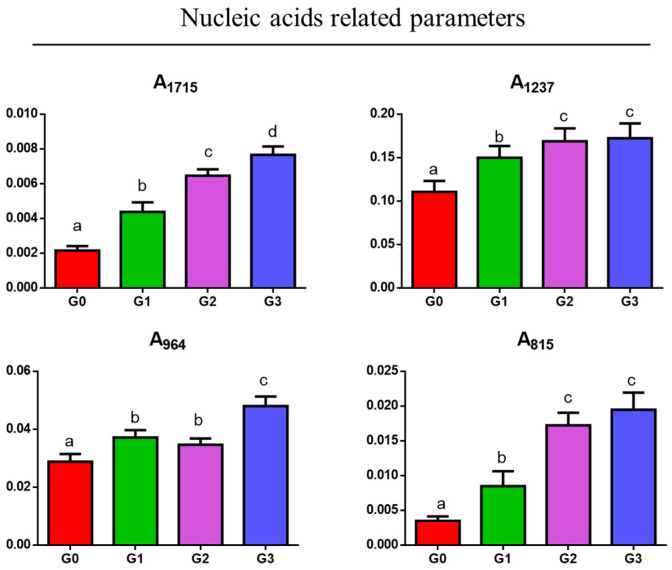
Statistical analysis of the spectral parameters related to the relative amount of nucleic acids: A_1715_, A_1237_, A_964_, and A_815_. Data are presented as mean ± S.D.; different letters indicate statistically significant differences among groups (one-way ANOVA and Tukey’s multiple comparison tests; *p* < 0.05).

For associating these spectral changes to a semiquantitative evaluation, IR data were submitted to univariate analysis. As regards nucleic acids (Figure 4), the following spectral parameters were calculated: A_1715_, calculated as the ratio between the area of the peak at 1715 cm^−1^ and the area of the 1800–750 cm^−1^ range; A_1237_, calculated as the ratio between the area of the peak at 1237 cm^−1^ and the area of the 1800–750 cm^−1^ range; A_964_, calculated as the ratio between the area of the peak at 964 cm^−1^ and the area of the 1800–750 cm^−1^ range; and A_815_, calculated as the ratio between the area of the peak at 815 cm^−1^ and the area of the 1800–750 cm^−1^ range. A statistically significant increase in all these parameters is observed (*p* < 0.05) going from G0 to G3, suggesting an increase in the inflammatory infiltrate.

**Figure 5:**
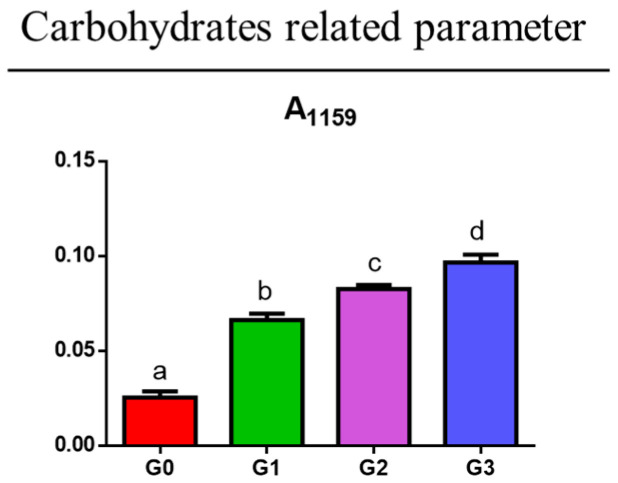
Statistical analysis of the spectral parameter A_1159_ related to the relative amount of carbohydrates. Data are presented as mean ± S.D.; different letters indicate statistically significant differences among groups (one-way ANOVA and Tukey’s multiple comparison tests; *p* < 0.05).

A similar trend is also shown by carbohydrates, as displayed by the increase in the parameter A_1159_ (*p* < 0.05), calculated as the ratio between the area of the peak at 1159 cm^−1^ and the area of the 1800–750 cm^−1^ range (Figure 5).

**Figure 6:**
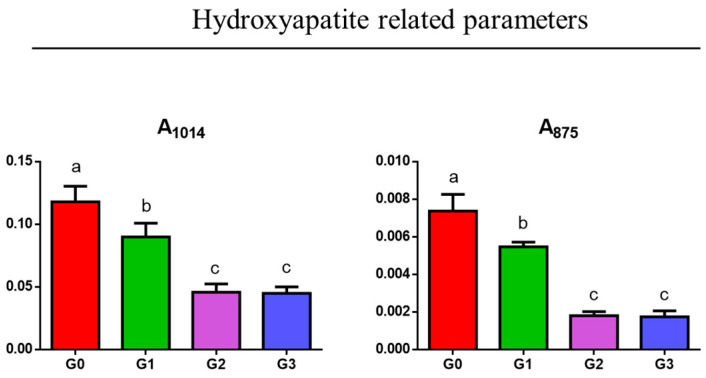
Statistical analysis of the spectral parameters related to the relative amount of hydroxyapatite: A_1014_ (inorganic phosphates), and A_875_ (inorganic carbonates). Data are presented as mean ± S.D.; different letters indicate statistically significant differences among groups (one-way ANOVA and Tukey’s multiple comparison tests; *p* < 0.05).

As regards the relative amount of hydroxyapatite, the following parameters were analyzed: A_1014_, calculated as ratio between the area of the peak at 1014 cm^−1^ and the area of the 1800–750 cm^−1^ range (representative of the inorganic phosphates), and A_875_, calculated as ratio between the area of the peak at 875 cm^−1^ and the area of the 1800–750 cm^−1^ range (representative of the inorganic carbonates). The highest values of both these ratios were observed in G0 (*p* < 0.05), while the lowest ones were in G2 and G3 groups, which displayed similar values to each other (*p* > 0.05); G1 presented intermediate values (*p* < 0.05) (Figure 6).

**Figure 7:**
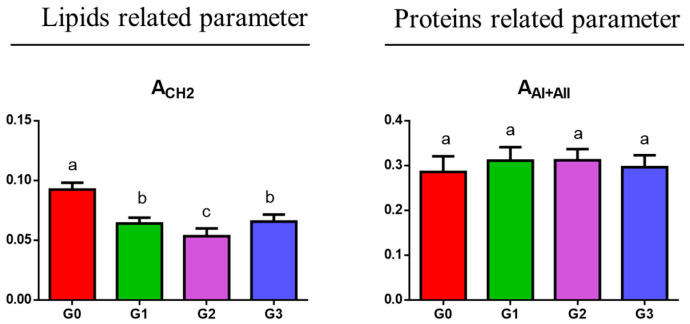
Statistical analysis of the spectral parameters related to the relative amount of lipids (A_CH2_), and proteins (A_AI+II_). Data are presented as mean ± S.D.; different letters indicate statistically significant differences among groups (one-way ANOVA and Tukey’s multiple comparison tests; *p* < 0.05).

Finally, two additional parameters related respectively to the relative amount of lipids and proteins were investigated: A_CH2_ (calculated as the ratio between the areas of the 3050–2800 cm^−1^ and 1800–750 cm^−1^ ranges) and A_AI+AII_ (calculated as the ratio between the sum of the areas of the Amide I and II bands of proteins and the area of the 1800–750 cm^−1^ range). The statistical analysis (displayed in Figure 7) evidenced: (i) a decrease in the relative amount of lipids going from G0 samples to G1, G2, and G3 ones (*p* < 0.05), these latter showing almost similar values (*p* > 0.05); (ii) no differences in proteins, which appeared approximately the same among groups (*p* > 0.05).

## 4. Discussion

Physiological RR in De is a dynamic and complex process that alternates periods of quiescence and activity, essential for the normal replacement of De with permanent teeth. To date, only a few studies have analyzed the biological features of this process, but none has provided information on the changes that occur in the dental pulp. Hence, to the best of the authors’ knowledge, this is the first study that gives information on the macromolecular composition and modifications of the dental pulp in relation to the degree of RR. The analysis has been carried out by using ATR-FTIR spectroscopy, an analytical tool able to provide the molecular characterization of biological samples in terms of the relative amount and composition of proteins, lipids, carbohydrates, nucleic acids, and phosphate groups [18].

It is known that, in the initial RR stages (corresponding to the G1 group), dystrophic calcification [25] and an increase in macrophages and T-lymphocytes with respect to stage G0 could be observed [2]. When the RR of De nears completion (corresponding to the G3 group), inflammatory cells infiltrate, and vascularization into the pulp tissues increases [26,27,28,29]. These findings are in accordance with our results; indeed, the analysis of the spectral region related to DNA indicates an increasing number of cells moving from G0 to G3. This could be attributed to the rise of the inflammatory infiltrate in the dental pulp, which is essential for modulating resorption activity by influencing odontoclast differentiation and function [30]. In fact, odontoclasts, multinucleated cells, are responsible for the degradation of cementum and dentin by resorbing the mineralized matrix through the secretion of acids and proteolytic enzymes and leaving resorption lacunae along the root surface [30]. Initial stages of RR (corresponding to G1 and G2) are characterized by active odontoclast recruitment and activity, whereas in later stages (G3), odontoclast numbers decrease and resorption slows down, possibly due to reduced signaling from surrounding tissues [31]. However, mainly due to the limited number of studies on this topic, there is disagreement in the literature as regards the presence of odontoclasts during the G3 stage: according to Eronat et al., there is a high number of odontoclasts [25], while other authors suggested that odontoclasts cannot be found [6,32].

Concomitant with the inflammatory infiltration, vascular changes occur within the pulp during RR [30]. Angiogenesis leads to increased vascularization, particularly in areas adjacent to resorption sites. Nevertheless, advanced stages of RR lead to a decline in pulp vitality, with widespread apoptosis of pulp cells, including odontoblasts [3], as indicated by the decrease, from G2 to G3, of the peak attributable to phosphate groups, related to the hydroxyapatite produced by these cells [3]. Thus, according to our results, in the stages, G2 and G3, inactive and degenerated odontoblasts could be present. On the contrary, during the early stages of RR (G0 and G1), the relative amount of hydroxyapatite (A_1014_ and A_875_) is represented, and it could be likely due to the production of dentin by odontoblasts [33,34].

The evaluation of CH_2_ groups confirms these findings since their reduction from G0 to G3 could be associated with the rupture of the plasma membrane of the odontoblasts, which undergo apoptosis [35]. Noteworthy, Rodrigues et al. proposed that apoptosis might be involved in the elimination of dental pulp during physiological RR [36]; in fact, by studying the apoptotic pathway, they suggested that apoptosis of dental pulp cells in this physiological process was more likely to occur through the activation of caspase-3 via the intrinsic mitochondrial pathway. Another important aspect is represented by the increase in carbohydrates during the RR process. In particular, it could be related to a decrease in cell activity due to the activation of glycogen synthase kinase-3 and, thereby, an increase in glycogen levels [37].

All the results here reported demonstrate, for the first time, that it is possible to assign specific spectral and, hence, molecular features to the dental pulp at each stage of the rizalysis, letting reject the null hypothesis. In fact, both multivariate and univariate analyses highlighted that the molecular pattern of G0 is completely different from that of G3, while G1 and G2 appear to have some spectral similarities to each other.

The macromolecular changes evidenced in the dental pulp in relation to the different degrees of RR represent also an important aspect in the therapeutic choice for deciduous teeth. In fact, premature loss of primary teeth can lead to orthodontic issues (e.g., crowding, ectopic eruption, malocclusion), speech problems, and psychosocial challenges, particularly in the case of anterior tooth loss [38]. Thus, knowing in depth the macromolecular changes that occur in the dental pulp at different stages of RR allows clinicians to carry out the most suitable and predictable treatment to achieve therapeutic success.

The results obtained in this study on the mechanisms involved in the physiological rizalysis of De can be summarized as follows:

- ATR-FTIR could represent a reliable technique that provides only a single analysis, and, on a few number of samples, the complete characterization of the organic and inorganic components of the dental pulp;
- ATR-FTIR analysis highlighted that each degree of RR corresponds to a defined spectral fingerprint of the dental pulp, associated with the relative amount and composition of lipids, carbohydrates, DNA, RNA, and phosphates;
- this physiological process is characterized by an increase in the relative amount of nucleic acids and carbohydrates from G0 to G3, and by a contemporary decrease in lipids and of phosphates and carbonates in hydroxyapatite.

## 5. Conclusions

This preliminary study confirms the importance of basic research for a better understanding of the physiological steps occurring in De root resorption and to fill some gaps in the clinical knowledge regarding the treatment of De teeth. Moreover, it offers a new point of view for the evaluation of the dental pulp using ATR-FTIR spectroscopy, which turned out to be a powerful and reliable analytical tool for quick and reliable characterization of biological samples at the molecular level, providing insights into the relative composition of proteins, lipids, carbohydrates, nucleic acids, and phosphate groups, without requiring sample preparation.

Hence, this study could represent the starting point for future research aiming to develop specific and more predictable clinical treatments for each stage; in fact, it is important for clinicians to understand pulp composition and biology and how this may affect their treatment decisions. These findings could be also important in the field of forensic dentistry, since the specific spectral features of the dental pulp can indicate the degree of rizalysis, thus allowing clinicians to identify the age of the subject.

## Figures and Tables

**Table 1:** Experimental groups are divided according to the degree of root resorption (RR).

Groups	Dental Pulps	Degree of RR
G0	7	No RR
G1	9	RR less than 1/3 of the root length
G2	10	RR not exceeded 2/3 of the root length
G3	10	RR more than 2/3 of the root length

**Table 2:** List of the most meaningful IR peaks, together with the associated vibrational mode and the biological meaning.

Peak Position (Wavenumbers, cm^−1^)	Vibrational Mode and Biological Meaning
~2920 cm^−1^ and ~2853 cm^−1^	Stretching of CH_2_ groups in lipid alkyl chains
~1742 cm^−1^	Stretching of C=O ester moiety in triglycerides and fatty acids
~1715 cm^−1^	Stretching of C=O in nucleic acids
~1651 cm^−1^, ~1628 cm^−1^, ~1540 cm^−1^, and ~1513 cm^−1^	Stretching of C=O, stretching of C=N, and bending of N-H in the peptide bond (Amide I and Amide II bands of proteins)
~1460 cm^−1^ and ~875 cm^−1^	Stretching of carbonate groups in hydroxyapatite
~1452 cm^−1^	Bending of CH_2_ groups in lipid alkyl chains
~1399 cm^−1^	Stretching of COO- moiety in amino acids
~1301 cm^−1^	Bending of N-H bond in nucleic acids
~1237 cm^−1^ and ~1086 cm^−1^	Asymmetric and symmetric stretching of organic phosphates in nucleic acids
~1159 cm^−1^	Stretching of C-OH groups in carbohydrates
~1122 cm^−1^	Symmetric phosphodiester stretching in RNA
~1014 cm^−1^	Stretching of inorganic phosphates in hydroxyapatite
~964 cm^−1^	Backbone vibration in DNA
~815 cm^−1^	Deformation of C-H in DNA

## Data Availability

The original contributions presented in this study are included in the article. Further inquiries can be directed to the corresponding authors.
